# Atypical Presentation of Cat-Scratch Disease in an Immunocompetent Child with Serological and Pathological Evidence

**DOI:** 10.1155/2014/397437

**Published:** 2014-12-28

**Authors:** Serkan Atıcı, Eda Kepenekli Kadayıfcı, Ayşe Karaaslan, Muhammed Hasan Toper, Cigdem Ataizi Celikel, Ahmet Soysal, Mustafa Bakır

**Affiliations:** ^1^Department of Pediatrics and Division of Pediatric Infectious Diseases, Marmara University Medical Faculty, Pendik Training and Research Hospital, Fevzi Cakmak Mah. Mimar Sinan Cad., Ust Kaynarca, Pendik, 34899 Istanbul, Turkey; ^2^Department of Pathology, Marmara University Medical Faculty, Pendik Training and Research Hospital, Fevzi Cakmak Mah. Mimar Sinan Cad., Ust Kaynarca, Pendik, 34899 Istanbul, Turkey; ^3^Marmara University Medical Faculty, Pendik Training and Research Hospital, Fevzi Cakmak Mah. Mimar Sinan Cad., Ust Kaynarca, Pendik, 34899 Istanbul, Turkey

## Abstract

Typical cat-scratch disease (CSD) is characterized by local lymphadenopathy following the scratch or bite from a cat or kitten. An atypical presentation which includes liver and/or spleen lesions is rarely reported in an immunocompetent child. Systemic CSD may mimic more serious disorders like malignancy or tuberculosis. Although a diagnosis is difficult to establish in systemic CSD, an early diagnosis and an appropriate treatment are important to prevent complications. *Bartonella henselae* is difficult to culture, and culture is not routinely recommended. Clinical, serological, radiological, and pathological findings are used for the diagnosis of CSD. Herein we present a case of systemic CSD presenting with hepatic mass in an immunocompetent child. The differential diagnosis is made by serological and pathological evidence. He was successfully treated with gentamicin (7.5 mg/kg) and rifampin (15 mg/kg) for six weeks.

## 1. Introduction

Cat-scratch disease (CSD) is an infectious disease caused by the* Bartonella henselae*. It is a small, curved, aerobic, slow-growing, fastidious, gram-negative, intracellular* Bacillus* that causes granulomatous inflammation of the tissue and it can be painted with silver stain [1].

CSD is usually associated with a previous history of exposure to cats or kittens. Although a history of scratch, bite, or licking from a cat or kitten is important, it is not necessary for the diagnosis. Typical CSD is characterized by local lymphadenopathy with or without skin rash and is usually self-limited. Systemic CSD may present in a more disseminated form which usually occurs in immunocompromised children. Atypical presentations which include hepatic and/or splenic lesions, osteomyelitis, discitis, granulomatous conjunctivitis, endocarditis, myocarditis, neuroretinitis, and encephalomeningitis may mimic more serious disorders such as malignancy and could be an important differential diagnostic problem [1, 2]. Isolated hepatic lesions of CSD are a rare clinical condition especially in an immunocompetent child.

We report the case of an immunocompetent child diagnosed as systemic CSD with serological and pathological evidence. He had multiple hepatic lesions. He was successfully treated with intravenous gentamicin and oral rifampin. The aim of this study was to report the management of hepatic CSD in an immunocompetent child.

## 2. Case Report

**Figure 1:**
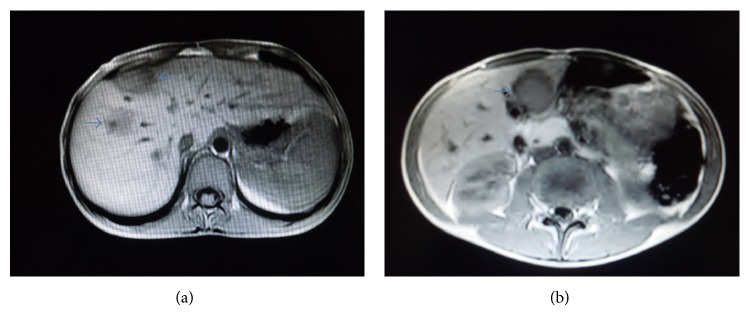
T1 (a) and T2 (b) weighted magnetic resonance imaging shows multiple lesions in the liver.

**Figure 2:**
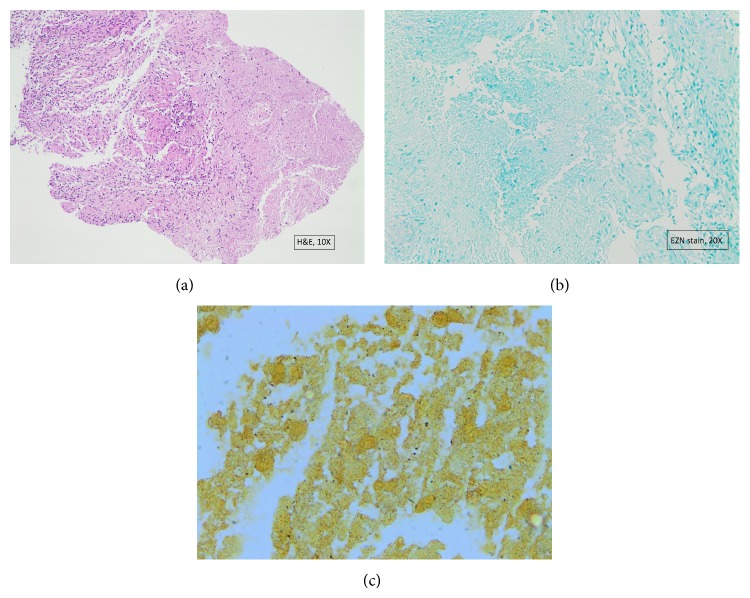
(a) The liver lesion is characterized by multiple granulomas hematoxylin and eosin ×10, (b) acid-fast Bacilli was not detected with EZN staining ×10, and (c)* Bartonella henselae* was demonstrated by Warthin-Starry silver stain ×10.

A 12-year-old boy was admitted to another hospital with 7-day history of fever, abdominal pain, headache, and weight loss. Empirical antibiotics therapy including ceftriaxone and clindamycin had been administered. After the abdominal ultrasound demonstrated multiple hypoechoic liver lesions, he was transferred to our department on the eleventh day of hospitalization. Fever and abdominal pain continued and he had lost 8 kilograms. He had a history of playing with a kitten. Physical examination revealed bilateral inguinal lymphadenopathy. There was not any scratch or papule on his skin. His past medical history was not consistent with any primary or secondary immunodeficiency or any other underlying disease. Laboratory findings included the following: white blood cell count, 10 400/mm^3^; hemoglobin, 11.9 g/dL; platelets, 385 000/mm^3^; C-reactive protein (CRP), 10.1 mg/dL; erythrocyte sedimentation rate (ESR), 57 mm in 1 h; aspartate aminotransferase (AST), 168 U/L; alanine aminotransferase (ALT), 67 U/L. Blood and urine cultures were negative. Serology of human immunodeficiency virus was also negative. Abdominal magnetic resonance imaging (MRI) showed multiple hepatic lesions (Figure 1). Cranial and thoracal imaging revealed no distinct abnormality. Serological analyses by indirect fluorescent antibody (IFA) method detected the presence of immunoglobulin (Ig) G and IgM antibodies to* Bartonella henselae* positive with a titer of 1 : 320 and 1 : 100, respectively. He was assessed to the pediatric immunology department with suspected immunodeficiency. Lymphocyte subset analysis, dihydrorhodamine 123 flow cytometry, and the serum immunoglobulins levels were normal. Ultrasound guided liver biopsy was performed. Histopathological analyses of the lesions showed granulomas surrounded with palisade histiocytes and non calcified necrosis with hematoxylin and eosin (H&E) staining (Figure 2(a)). Acid-fast Bacilli were not detected with Ehrlich-Ziehl-Neelsen (EZN) staining (Figure 2(b)). Warthin-Starry silver stain was positive which was compatible with CSD (Figure 2(c)).

He was treated with intravenous gentamicin (7.5 mg/kg) and oral rifampin (15 mg/kg) for six weeks. His symptoms were resolved and abnormal laboratory findings including elevated CRP, ESR, and liver transaminases were normalized after antibiotic therapy. Contrast enhanced abdominal MRI was repeated on 30th day of antibiotic therapy and showed that hepatic lesions had regressed.

## 3. Discussion

*Bartonella henselae* is the etiologic agent of cat-scratch disease and cats are the major reservoir for the bacteria. A study in our country determined* Bartonella henselae* IgG antibody seroprevalence was 18.8% in cats. Seropositivity was observed as 27.5% for stray cats. The seropositivity was 14.3% and 11.4% in outdoor and indoor domestic cats in Turkey, respectively [3]. History of scratch or bite from a cat or kitten is helpful for the diagnosis. Our patient had a history of playing with a kitten.

Although most patients with CSD typically presented with fever and lymphadenopathy, atypical clinical manifestations may be seen.Atypical clinical presentations of CSD include a broad spectrum of clinical syndromes ranging from prolonged fever of unknown origin to hepatosplenic, ocular, and neurological manifestations [4, 5]. Hepatosplenic disease is an unusual clinic presentation occurring in only 0.3% to 0.7% of patients, mostly in children [6]. Hepatic involvement is an uncommon clinical presentation in immunocompetent children. Patients with hepatic lesions present episodic abdominal pain with prolonged fever. Although liver enzymes are usually normal, erythrocyte sedimentation rate is often increased.

Abdominal imaging is an important diagnostic step in patients with suspected hepatosplenic CSD [4]. Ultrasonography (US), computed tomography (CT), or MRI may show multiple and variable size and shape lesions in the liver or spleen. Lesions are usually hypoechoic on US and hypoattenuated on CT. Multiple hepatic lesions may be due to lymphoma, histoplasmosis, granulomatous processes, and metastatic disease [7, 8].

*Bartonella henselae* is difficult to culture, and culture is not routinely recommended. Clinical, serological, radiological, and pathological findings are used for the diagnosis of CSD. In history, the exposure to animals, especially cats, is important medical information to suspect from CSD [9]. Serological testing for* Bartonella henselae* antibodies is the most cost-effective diagnostic modality with IFA to be most frequently used [4, 10]. Granulomatous inflammation is not specific to CSD, so other granulomatous diseases such as tuberculosis should be considered. Although tuberculosis is still common in Turkey, isolated hepatic lesions are unusual. We have investigated for tuberculosis and ruled out histopathological findings and laboratory tests including IFN-gamma releasing assay (QuantiFERON-TB), EZN staining, and mycobacterial culture of gastric lavage fluids. Acid-fast* Bacillus* was not detected on liver biopsy with Ehrlich- Ziehl-Neelsen staining but* Bartonella henselae* has been demonstrated as short rods by using Warthin-Starry silver stain (Figure 2(c)). Previous history of contact with kitten, the positive serology for* Bartonella henselae*, and radiological and pathological findings were used for the diagnosis of our patient.

Systemic CSD has a high morbidity rate in immunocompromised children. There is no consensus about the type of antimicrobial drug and the duration of the therapy for the diagnosis of systemic CSD in an immunocompetent child [11]. Rifampin, trimethoprim-sulfamethoxazole, gentamicin, macrolides, extended spectrum cephalosporins, and ciprofloxacin all have* in vitro* activity against* Bartonella henselae* [11, 12]. Arısoy et al. published a large series including 19 children with hepatosplenic cat-scratch disease and all patients were treated with one or more antibiotics including gentamicin (7.5 mg/kg), rifampin (15–20 mg/kg), and trimethoprim-sulfamethoxazole (10–12 mg/kg) for 10 to 21 days. Thirteen patients were treated with rifampin alone and 3 patients were treated with rifampin plus gentamicin or trimethoprim-sulfamethoxazole. Rifampin was proposed in the antimicrobial treatment of hepatosplenic CSD in that study [12]. We used a rifampin plus gentamicin combination therapy in our case for 6 weeks. His symptoms were resolved and the hepatic lesions regressed. A relapse was not determined during the follow-up.
